# Influence of Resilience and Optimism on Distress and Intention to Self-Isolate: Contrasting Lower and Higher COVID-19 Illness Risk Samples From an Extended Health Belief Model

**DOI:** 10.3389/fpsyg.2021.662395

**Published:** 2021-05-24

**Authors:** Sergio Cervera-Torres, Susana Ruiz-Fernández, Hendrik Godbersen, Lena Massó, David Martínez-Rubio, Sheila Pintado-Cucarella, Rosa M. Baños

**Affiliations:** ^1^Department Multimodal Interaction Lab, Leibniz-Institut für Wissensmedien (IWM), Tübingen, Germany; ^2^Department of Psychology, Valencian International University (VIU), Valencia, Spain; ^3^LEAD Research Network, Eberhard Karls University of Tübingen, Tübingen, Germany; ^4^FOM Hochschule für Oekonomie and Management, Essen, Germany; ^5^Psicoforma, Psychology Center, Valencia, Spain; ^6^Department of Psychology, Faculty of Health Sciences, Universidad Europea de Valencia, Valencia, Spain; ^7^Department of Psychology, Universidad de las Américas, Puebla, Mexico; ^8^Department of Personality, Evaluation and Psychological Treatment, Universidad de Valencia, Valencia, Spain; ^9^Centro de Investigación Biomédica en Red of Physiopathology of Obesity and Nutrition, Madrid, Spain

**Keywords:** COVID-19, resilience (psychological), dispositional optimism, distress, intention to self-isolate, health belief model, illness risk

## Abstract

The study investigated the influence of resilience and dispositional optimism on, first, emotional distress and, second, the intention to self-isolate, experienced by people with a lower and higher illness risk, during the lockdown imposed in Spain during the first COVID-19 wave. These effects were investigated against the background of the Health Belief Model (HBM). A convenience sample of *N* = 325 participants completed an online survey including an *ad-hoc* questionnaire measuring the HBM core factors: Perceived health threat (susceptibility and severity of getting infected), and perceived quarantine benefits and costs. Self-efficacy and perceived social pressure were also measured. Based on reviews regarding pandemic outbreaks, quarantine benefits were conceptualized as the perceived effectiveness and solidary contribution of self-isolating in line with the quarantine protocols. Quarantine “psychosocial” costs were conceptualized as a composite of perceived boredom, loneliness, and economic concerns. Findings revealed an asymmetrical pattern of results so that (i) people at higher risk were more distressed by the perceived severity of getting infected whereas people at lower risk were more distressed by the psychosocial costs. Moreover, (ii) resilience and optimism were more “protective” against distress within the lower and higher risk groups, respectively. In addition, (iii) quarantine benefits and self-efficacy promoted the intention to self-isolate within both groups. However, (iv) optimism hindered such intention. This finding is discussed in the light of links between dispositional optimism and optimistic bias; the underestimation of experiencing negative events, which can relax the perceived health risk. Based on these findings, communication campaigns should prioritize information about the effectiveness of the implemented preventive behaviors rather than the costs of not implementing them, and be cautionary in encouraging excessive optimism.

## Introduction

The coronavirus outbreak (COVID-19) has triggered a sanitary alarm worldwide, promoting preventive health behaviors such as frequent hand washing, use of sanitary masks, and the so-called social distancing measures. Compliance with these measures has been deemed pivotal to curb infection spreading and, in turn, mortality rates of people at higher risk of illness and death, i.e., usually people of advanced age and/or with chronic health conditions (e.g., Noor and Islam, 2020).

According to the Health Belief Model (HBM; Rosenstock, 1974), implementing preventive health behaviors (or behavior intention as a proxy; cf. Fishbein et al., 2001) depends on perceiving (1) health threat, which is composed of susceptibility and severity (i.e., the perceived likelihood and seriousness of being affected by a disease such as COVID-19), and (2) assessing the costs and benefits of a preventive behavior. For example, the perceived costs in terms of loneliness, boredom, and economic concerns associated with self-isolating during pandemic quarantines (cf. Brooks et al., 2020; Wolff et al., 2020) or, in contrast, conceiving the self-isolation as the contribution to an “effective and civic cause” that can reduce spreading the virus (cf. Clark et al., 2020; Webster et al., 2020). Moreover, (3) individual characteristics (e.g., gender or age), (4) cues to action (e.g., perceived social pressure; cf. Godbersen et al., 2020), and (5) self-efficacy (i.e., perceived control or confidence to implement a course of action; Bandura, 1993) are also considered (for a review see Champion and Skinner, 2008).

Compliance with preventive health measures, however, has also been associated with affective processes such as emotional distress (e.g., Siebenhaar et al., 2020) during the COVID-19 outbreak. An important point addressed here is that psychological factors supporting mental well-being, thus, being protective against distress, could also be associated, to some extent, with compliance behavior. This is the case of resilience, which can be understood as the ability to adaptively coping or bouncing back from adversities (e.g., Fletcher and Sarkar, 2013) and dispositional optimism (i.e., the tendency to think in terms of future positive outcomes; e.g., Carver and Scheier, 2014). Both variables have been shown positively associated with mental well-being (e.g., Kimhi et al., 2020; Robles-Bello et al., 2020) and preventive behaviors (e.g., Pasion et al., 2020; Yildirim and Arslan, 2020). Against this backdrop, the aim of the present study is to build upon the HBM to deepen such associations in the particular context of the lockdown imposed in Spain during the first COVID-19 wave.

### Investigating Resilience, Optimism, and Emotional Distress During the Spanish Lockdown

Officially, a lockdown in terms of home-quarantine was declared in Spain on 14th March 2020. Its total extension was initially uncertain and was prolonged for approximately one and a half months. People were required to self-isolate at home. Only essential activities were permitted (e.g., buying groceries and medicines, receiving medical assistance or in some cases, going to work). As covered by the media (cf. Tejedor et al., 2020), however, many people found it difficult to strictly comply with the mobility restriction due to different needs (e.g., need of exercising outdoors or visiting relatives). Furthermore, variability in the intensity of distress has been reported during the quarantine in Spain as linked to, first, economic concerns, which is not surprising since the economic situation in Spain was just recovering from the financial crisis of 2009. Moreover, second, emotional distress was also associated to the negative perception of own health, particularly regarding people at higher illness risk (Rodríguez-Rey et al., 2020). It is pivotal to highlight that people at higher illness risk, although they can feel very stressed by the possibility of catching the virus (e.g., Kontoangelos et al., 2020), can face problems to comply with social distancing measures, especially self-isolation (e.g., Daoust, 2020).

In the light of the foregoing, the present study explores, first, the protective role of resilience and optimism against emotional distress and also the role of these variables on the intention to self-isolate during the quarantine in Spain. Additionally, the study raises the question of whether people at lower and higher COVID-19 illness risk show a similar pattern of effects.

## Methods

### Data Collection

**Figure 1:**
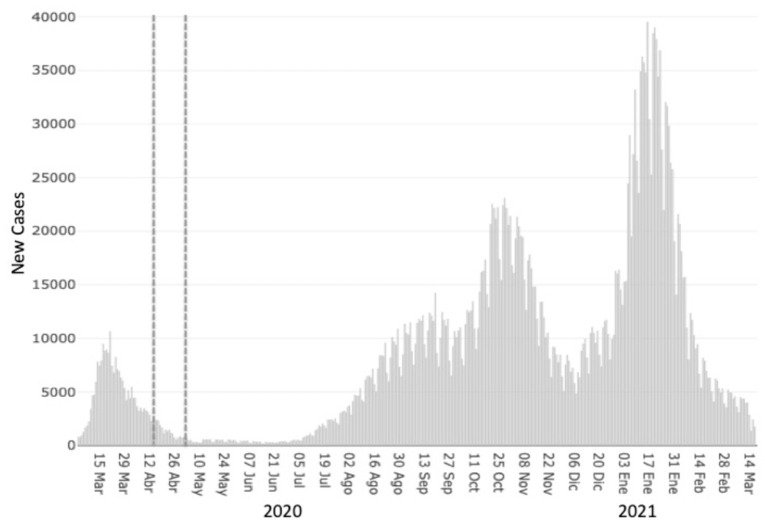
Graphical representation of the COVID-19 pandemic in Spain. Dashed lines represent the period when the data of this study was collected. Figure slightly adapted from the Spanish Ministry of Health website. https://cnecovid.isciii.es/covid19/#ccaa.

The study was conducted *via* SoSci Survey (https://www.soscisurvey.de/en/index). Data were collected from April 15th until May 3rd 2020, ~1 month after the lockdown started. The situation at these two-time points was of 2,385 COVID-19 new reported cases (592 deceases) and 461 cases (273 deceases), respectively (see Figure 1).

Participants were provided with information about the study, including data protection and anonymity, approximate time of survey completion (15–20 min), and informed consent. Moreover, an option to leave the survey and delete the data was made clearly available. Participants did not receive any compensation for the participation. Data collection was in accordance with the 1964 Helsinki declaration and its later amendments.

### Participants

The sampling was based on a non-probabilistic snowball approach (cf. Baltar and Gorjup, 2012). From the 506 participants that initiated the survey, *N* = 325 (68.9% women) completed the necessary information, which compose the final sample of the study. Participants were in the age range of 19–75 (*M* = 43.5, *SD* = 13.1) and came from 15 out of the 16 autonomous regions of Spain. In line with the guidelines of the Spanish Ministry of Health, participants of 60 years old onwards or reporting a chronic health condition were considered as a group at higher COVID-19 illness risk. This group nearly represented 1/3 (28.9%) of the total sample (see Table 1).

**Table 1:** Demographics related to the COVID-19 illness risk.

	**COVID-19 illness risk**	
	**Lower (*n* = 231)**	**Higher (*n* = 94)**
***Gender***		
Woman	163 (70.5%)	61 (64.9%)
Man	68 (29.4%)	33 (35.1%)
***Age***		
Range	19–59	29–75
*M* (SD)	39.4 (10.7)	53.7 (12.6)
Chronic Condition	–	62 (65.9%)

### Variables

#### Emotional Distress

The 4-item version of the Patient Health Questionnaire (PHQ-4; Kroenke et al., 2009) was used in Spanish translation (cf. Diez-Quevedo et al., 2001; Rodríguez-Muñoz et al., 2020). The instrument monitors symptoms of anxiety and depression perceived during the last 2 weeks (e.g., “not being able to stop or control worrying”). Responses ranged from 0 (not at all) to 3 (nearly every day). Moreover, the instrument also proposes a composite score defining normal (0–2), mild (3–5), moderate (6–8), and severe (9–12) mental health levels (Chronbach's α = 0.747). In this study, we conceptualized this composite score as emotional distress (cf. Daly and Robinson, 2020).

#### Intention to Self-Isolate

Two items were generated to evaluate this variable. Responses ranged from 1 (“strongly disagree”) to 7 (“strongly agree”). The first addressed the commitment to self-isolate in line with the quarantine protocol. Therefore, higher responses indicated a higher commitment to self-isolate at home. In contrast, the second item addressed the idea that withstanding the quarantine required leaving home more than the strictly necessary, thus, going against the quarantine protocol. In this case, higher responses indicated a greater need of leaving home. As expected, both items were negatively correlated (*r*_S_ = −0.34; *p* < 0.001). A composite score, which was conceptualized as the *intention to self-isolate*, was calculated by adding the reversed coded second item (“leaving home”) to the first item (“commitment”). In contrast, a composite score, which was conceptualized as the *discrepancy to self-isolate* in the sense of the need to leave home, was calculated by adding the reversed coded first item (“commitment”) to the second item (“leaving home”).

#### HBM Factors

A brief 10-item questionnaire was generated *ad-hoc* to evaluate the core HBM factors: perceived susceptibility, severity, quarantine benefits, and costs (see Table 2). Susceptibility and severity items evaluated the perceived likelihood of getting infected by COVID-19 and the related worries and fears. Based on a recent review on pandemic quarantines by Webster et al. (2020), quarantine benefits were conceptualized as the perceived effectiveness and contribution to a common civic cause. On the other hand, quarantine costs were conceptualized as a composite of perceived loneliness, boredom, and economic concerns (cf. Brooks et al., 2020). All responses ranged from 1 (“strongly disagree”) to 7 (“strongly agree”). A confirmatory factor analysis (CFA; Brown, 2015) supported the four HBM factors (see Supplementary Material).

**Table 2:** Items of the HBM questionnaire used in the study.

**Susceptibility**
Item 1. Es probable que me contagie por coronavirus en las próximas semanas/meses. *It is likely that I will get infected with the coronavirus in the next few weeks/months*.
Item 2^*^. Contagiarme por coronavirus es algo que no he considerado. *I did not consider the possibility of catching the coronavirus*.
**Severity**
Item 3. Si percibo a alguien cerca de mi, me siento vulnerable a un contagio por coronavirus. *If I perceive someone near me, I feel vulnerable to a coronavirus infection*.
Item 4. Me preocupa mucho la posibilidad de poder contagiarme por coronavirus.*I am very worried about the possibility of catching the coronavirus*.
Item 5. Temo no poder despedirme de un ser querido si llegara a contagiarme por coronavirus.*I am afraid I won't be able to say goodbye to a loved one if I get infected with the coronavirus*.
**Quarantine Benefits**
Item 6. Respetando la cuarentena evito contagiarme y contagiar a otras personas por coronavirus. *I avoid catching and spreading the coronavirus to others by adhering to the quarantine*.
Item 7. Respetando la cuarentena contribuyo a un bien social común. *I contribute to a common social benefit by adhering to the quarantine*.
**Quarantine Costs**
Item 8. Siento más soledad desde que comenzó la cuarentena. *I experience more loneliness since the quarantine started*.
Item 9. Desde que comenzó la cuarentena, siento más aburrimiento. *I am more bored since the quarantine started*.
Item 10. Desde que comenzó la cuarentena siento mucha preocupación por mi situación económica. *I am very concerned about my economic situation since the quarantine started*.

* *Inverted item*.

Two additional items were generated to assess perceived social pressure to self-isolate during the quarantine (i.e., the perception that, in general, most people expect one to self-isolate in line with the quarantine protocol) and to assess self-efficacy in terms of perceived capability to withstand the quarantine for uncertain time.

#### Resilience

The Brief Resilient Coping Scale (BRCS; Sinclair and Wallston, 2004) was used in its Spanish version (Limonero et al., 2014; Chronbach's α = 0.76). It is a 4-item scale, in which the responses range from 1 (“does no describe me at all”) to 5 (“describes me very well”).

#### Dispositional Optimism (DO)

DO was measured by the Revised Life Orientation Test (LOT-R; Scheier et al., 1994) in its Spanish version (Ferrando et al., 2002). The scale is composed of 10 items, 4 of which are fillers, 3 evaluate optimism, and 3 pessimism. Responses ranged from 0 (“strongly disagree”) to 4 (“strongly agree”).

### Statistical Analyses

The analyses were run in IBM SPSS Statistics v21. The descriptive analyses and comparisons between the two risk groups were calculated in the first step. In the second step, correlation analyses were performed to inspect associations between the variables. Multiple regression analyses were performed in a third step to examine the influence of the HBM factors, resilience, and optimism on distress and the intention to self-isolate.

## Results

### Descriptive Analyses

As shown in Table 3, the group at higher illness risk associated a greater severity to COVID-19 than the group at lower illness risk. The other variables did not show significant differences between the two groups.

**Table 3:** Descriptive analyses of the variables in the study.

	***M* (SD)**	**Min–Max**	***M*%**	**S**	**K**	***M* (SD) Lower risk (*n* = 231)**	***M* (SD) Higher risk (*n* = 94)**	***t***	***p***
Distress	3.84 (2.45)	0–12	32.0%	4.86	2.56	3.77 (2.23)	4.01 (2.91)	−0.73	0.47
S. Anxiety	2.04 (1.45)	0–6	34.0%	4.83	0.42	1.96 (1.39)	2.22 (1.54)	−1.39	0.16
S. Depression	1.80 (1.34)	0–6	30.0%	5.01	2.01	1.81 (1.24)	1.79 (1.54)	0.10	0.92
I. Self-isolate	11.30 (2.60)	2–14	80.9%	−6.34	0.04	11.16 (2.64)	11.76 (2.46)	−1.87	0.061
Susceptibility	9.07 (2.85)	2–14	64.8%	−1.01	−1.98	9.21 (2.86)	8.72 (2.81)	1.40	0.16
Severity	13.69 (4.44)	3–21	65.2%	−2.68	1.72	13.21 (4.54)	14.88 (3.94)	−3.32	0.001
Q. Costs	11.47 (3.70)	3–21	54.6%	0.71	0.93	11.53 (3.58)	11.32 (4.00)	0.48	0.63
Q. Benefits	13.19 (1.70)	2–14	94.2%	−22.44	42.54	13.17 (1.80)	13.25 (1.43)	−0.42	0.68
Social pressure	5.88 (1.25)	1–7	84.0%	−11.02	10.87	5.88 (1.30)	5.88 (1.12)	0.029	0.97
Self-efficacy	3.85 (1.94)	1–7	55.0%	−0.48	4.29	3.84 (1.95)	3.88 (1.92)	−0.18	0.85
Resilience	14.83 (2.57)	5–20	74.1%	2.28	1.11	14.85 (2.53)	14.77 (2.66)	0.27	0.78
D. Optimism	12.50 (2.93)	4–23	54.3%	1.44	2.27	12.46 (2.90)	12.61 (3.02)	−0.41	0.68

*Standardized Skewness values |S| ≥ 3 and Kurtosis ≥ 7 |K| are considered as departures of normal distribution (cf. Kim, 2013). Bonferroni correction for multiple comparisons was applied. Percentages represent average proportions with regard to the maximum score of each variable. Distress is reported as a composite score of anxiety and depression symptoms (S)*.

**Figure 2:**
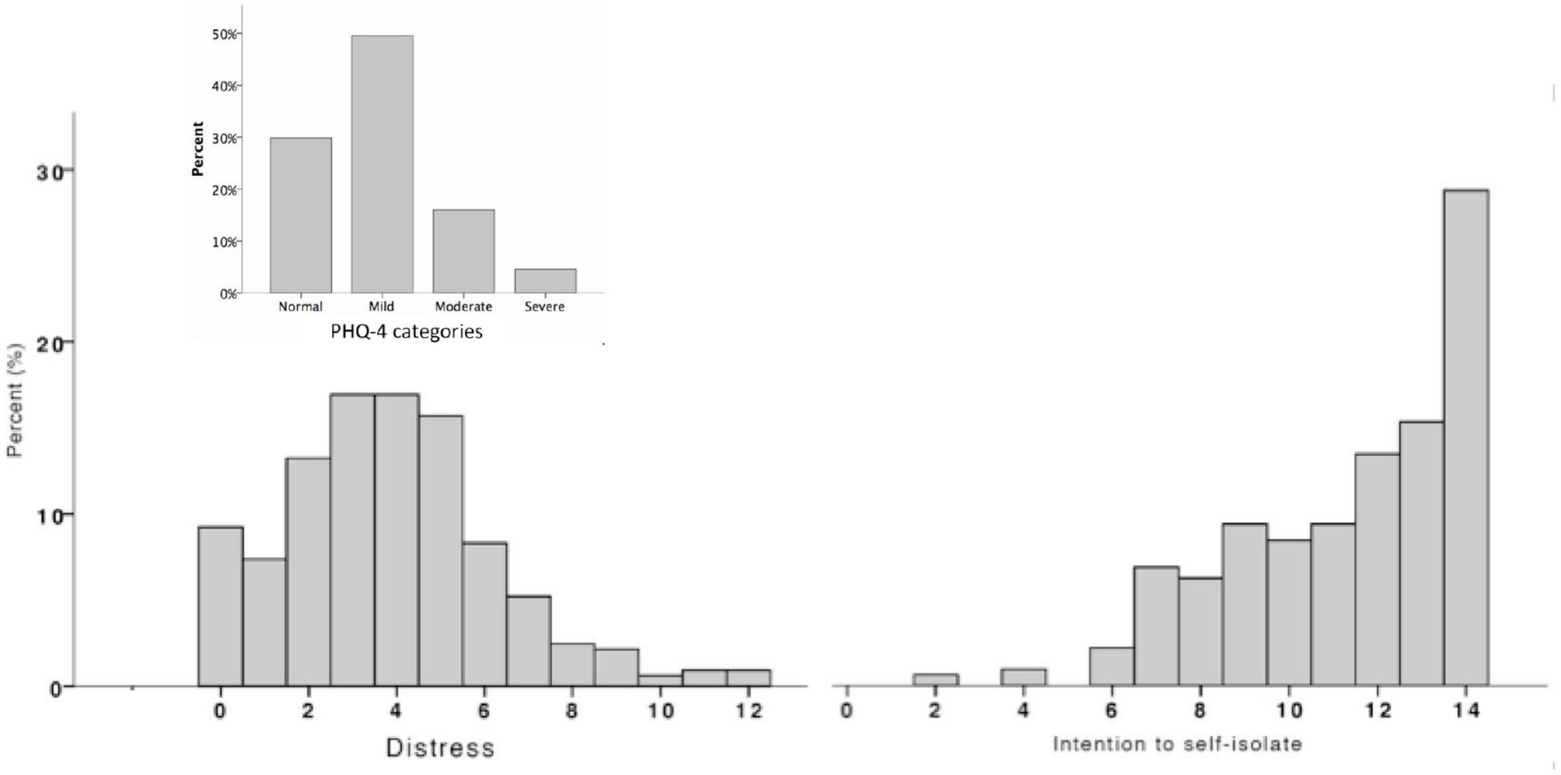
Left portion shows frequencies of distress with PHQ-4 categories (upper part). Right portion shows frequencies of intention to self-isolate.

Levels of distress (and symptoms of anxiety and depression, independently) were rather mild, on average. Scores based on the PHQ-4 mental health categories indicated that 29.8% of participants showed normal symptoms of distress and half of participants reported mild symptoms (49.5%). The rest, however, showed moderate (16%) and severe (4.6%) symptoms (see Figure 2).

Data also shows that, in general, the perceived quarantine costs were moderate and that participants highly agreed on the perceived quarantine benefits (94.2%). A closer inspection of the composite scores related to quarantine costs and benefits revealed that the group at lower illness risk perceived greater economic concerns. Perceived loneliness, boredom, quarantine effectiveness, and sense of solidarity did not show significant differences between the groups (see Supplementary Table 1).

The agreement with the perceived quarantine benefits is also in line with the reported high intention to self-isolate (80.9%). Yet, the variability of the intention to self-isolate showed that not every individual was totally prone to strictly stick to the mobility restriction, which might be associated with the moderate perceived capability to withstand the quarantine for uncertain time (55%).

Importantly, the risk groups did not show significant differences concerning resilience, which average level was quite high (74.1%), and dispositional optimism, which average level was rather moderate (54.3%).

### Correlation Analyses

Spearman rank correlations in Table 4 indicate, first, that neither distress nor symptoms of anxiety and depression yielded a significant association with the intention to self-isolate.

**Table 4:** Spearman rank correlations of the variables in the study.

	**1**	**2**	**3**	**4**	**5**	**6**	**7**	**8**	**9**	**10**	**11**	**12**
1. Distress	–											
2. S. Depression	0.843^**^	–										
3. S. Anxiety	0.875^**^	0.501^**^	–									
4. I. Self-isolate	−0.007	−0.049	0.055	–								
5. Susceptibility	0.198^**^	0.134^*^	0.203^**^	0.123^*^	–							
6. Severity	0.267^**^	0.140^*^	0.328^**^	0.066	0.068	–						
7. Q. Costs	0.403^**^	0.375^**^	0.329^**^	−0.035	0.082	0.165^**^	–					
8. Social pressure	0.069	0.031	0.089	0.309^**^	0.203^**^	0.023	−0.031	–				
9. Q. Benefits	0.056	−0.013	0.118^*^	0.314^**^	0.131^*^	0.182^**^	−0.042	0.323^**^	–			
10. Self-efficacy	−0.262^**^	−0.262^**^	−0.211^**^	0.292^**^	0.011	−0.051	−0.257^**^	0.175^**^	0.129^*^	–		
11. Resilience	−0.308^**^	−0.285^**^	−0.268^**^	0.002	−0.090	−0.163^**^	−0.120^*^	0.063	0.045	0.167^**^	–	
12. D. Optimism	−0.050	−0.068	−0.024	−0.160^**^	−0.144^**^	0.105	0.002	−0.049	−0.027	0.048	0.111^*^	–

** *p <0.01*;

* *p <0.05*.

Moreover, second, distress and intention to self-isolate were almost completely linked to a different set of variables. While quarantine costs yielded the highest association with distress (i.e., the higher the perceived costs, the higher the distress), quarantine benefits did so with the intention to self-isolate (i.e., the higher the perceived benefits, the higher the intention to self-isolate). Self-efficacy was associated with both, lower distress and higher intention to self-isolate, in a comparable manner.

Importantly, resilience played the greatest “protective” role in mental health, being associated with lower distress, weaker symptoms of depression and anxiety, lower perceived severity, and lower perceived costs. Optimism, on the other hand, did not show a significant association with distress, including symptoms of anxiety and depression. Interestingly, optimism was associated with lower susceptibility of getting infected as well as the intention to self-isolate.

We used a multiple regression analysis to investigate if the result patterns held up in the lower and higher COVID-19 risk groups.

### Regression Analyses

A generalized multiple regression approach was used to investigate the extent to which the HBM factors, as well as, resilience and dispositional optimism, influenced distress and the intention to self-isolate. Results concerning symptoms of depression and anxiety are also reported (see Supplementary Table 2). Data modeling was set with normal distribution and a log-link. Due to its positively skewed distribution, intention to self-isolate was analyzed in terms of *discrepancy to self-isolate* as described before. Predictors were included in the model as *z*-scores. Gender and age were included as control covariates. Influential observations were inspected *via* Cook distances. Robust confident intervals were estimated *via* the Huber-White approach. Pseudo-*R*^2^ was calculated based on deviance residuals (see Table 5).

**Table 5:** Multiple regression analyses predicting distress and discrepancy to self-isolate.

	**Total sample**			**Lower illness risk**			**Higher illness risk**	
**Distress**	***Pseudo R***^****2****^ **=** **0.377**			***Pseudo R***^****2****^ **=** **0.425**			***Pseudo R***^****2****^ **=** **0.508**	
	***b***	**95% CI**	***b*_**%**_ (Δ*R*^**2**^)**	***b***	**95% CI**	***b*_**%**_ (Δ*R*^**2**^)**	***b***	**95% CI**	***b*_**%**_ (Δ*R*^**2**^)**
Gender	0.231	(−0.363, −0.100)^**^	20.7% (0.047)^**^	0.207	(−0.339, −0.076)^**^	18.7% (0.048)^**^	0.139	(−0.420, 0.143)	–
Age	−0.071	(−0.133, −0.010)^*^	6.9% (0.033)^**^	−0.032	(−0.090, 0.026)	–	−0.254	(−0.378, −0.129)^**^	22.4% (0.149)^**^
Susceptibility	0.075	(0.010, 0.140)^*^	7.8% (0.030)^**^	0.070	(0.006, 0.134)^*^	7.3% (0.042)^**^	0.113	(−0.034, 0.260)	–
Severity	0.120	(0.046, 0.194)^**^	12.8% (0.099)^**^	0.045	(−0.021, 0.112)	–	0.215	(0.114, 0.317)^**^	24.0% (0.188)^**^
Q. Costs	0.152	(0.089, 0.215)^**^	16.5% (0.116)^**^	0.186	(0.128, 0.244)^**^	20.4% (0.184)^**^	0.164	(0.057, 0.270)^**^	17.8% (0.057)^**^
Q. Benefits	−0.010	(−0.061, 0.040)	–	−0.009	(−0.049, 0.031)	–	0.033	(−0.090, 0.155)	–
Social pressure	0.024	(−0.040, 0.088)	–	0.044	(−0.017, 0.104)	–	−0.100	(−0.265, 0.066)	–
Self-efficacy	−0.064	(−0.125, 0.003)^*^	6.2% (0.025)^*^	−0.105	(−0.167, −0.043)^**^	10.0% (0.054)^**^	−0.009	(−0.131, 0.112)	–
Resilience	−0.148	(−0.192, −0.104)^**^	13.8% (0.096)^**^	−0.141	(−0.185, −0.096)^**^	13.1% (0.106)^**^	−0.118	(−0.241, −0.004)	–
D. Optimism	−0.023	(−0.091, 0.044)	–	0.023	(−0.034, 0.080)	–	−0.188	(−0.328, −0.049)^**^	17.2% (0.091)^**^
**Self-isolate (discrepancy)**	***Pseudo R***^**2**^ **=** **0.280**			***Pseudo R***^**2**^ **=** **0.258**			***Pseudo R***^**2**^ **=** **0.436**	
	***b***	**95% CI**	***b***_**%**_ **(Δ*****R***^**2**^**)**	***b***	**95% CI**	***b***_**%**_ **(Δ*****R***^**2**^**)**	***b***	**95% CI**	***b***_**%**_ **(Δ*****R***^**2**^**)**
Gender	−0.186	(0.074, 0.297)^**^	20.4% (0.026)^**^	−0.179	(0.047, 0.311)^**^	19.6% (0.026)^*^	−0.195	(−0.039, 0.428)	–
Age	0.017	(−0.031, 0.065)	–	0.052	(−0.005, 0.108)	–	0.014	(−0.117, 0.146)	–
Susceptibility	−0.011	(−0.069, 0.047)	–	−0.004	(−0.080, 0.071)	–	−0.051	(−0.175, 0.073)	–
Severity	−0.020	(−0.080, 0.040)	–	0.013	(−0.061, 0.087)	–	−0.088	(−0.188, 0.013)	–
Q. Costs	−0.026	(−0.081, 0.028)	–	−0.035	(−0.095, 0.024)	–	0.023	(−0.115, 0.161)	–
Q. Benefits	−0.138	(−0.170, −0.105)^**^	12.9% (0.102)^**^	−0.145	(−0.176, −0.114)^**^	13.5% (0.116)^**^	−0.166	(−0.208, −0.024)^*^	10.9% (0.056)^**^
Social pressure	−0.021	(−0.068, 0.026)	–	−0.003	(−0.058, 0.052)	–	−0.079	(−0.214, 0.056)	–
Self-efficacy	−0.190	(−0.247, −0.132)^**^	17.3% (0.113)^**^	−0.175	(−0.249, −0.101)^**^	16.0% (0.093)^**^	−0.177	(−0.311, −0.043)^**^	16.2% (0.107)^**^
Resilience	−0.003	(−0.061, 0.056)	–	0.005	(−0.062, 0.072)	–	−0.021	(−0.141, 0.099)	–
D. Optimism	0.117	(0.060, 0.173)^**^	12.4% (0.053)^**^	0.093	(0.024, 0.162)^**^	9.7% (0.033)^**^	0.122	(0.027, 0.235)^*^	14.0% (0.074)^*^

*p <0.1*;

* *p <0.05*;

** *p <0.01*.

#### Emotional Distress

The analyses revealed that, in general, women (vs. men) scored higher in distress. This result is in line with other studies reporting this effect for the Spanish population during the quarantine (Rodríguez-Rey et al., 2020). Furthermore, it was also showed by meta-analyses examining data from other countries during the COVID-19 outbreak (e.g., Wang et al., 2020). The analyses of the present study also revealed that emotional distress decreased by age.

Interestingly, our analyses revealed as well that women (vs. men) reported higher symptoms of anxiety within the group at lower risk but higher symptoms of depression within the group at higher risk, thus, reflecting asymmetries between the two groups.

The health threat components (i.e., susceptibility and severity) and quarantine costs predicted higher levels of distress, in general. However, inspecting in more detail the results concerning the lower and higher risk groups, revealed important differences. Specifically, within the lower risk group, the most important predictor of distress (and also symptoms of anxiety and depression) was the perceived quarantine costs (i.e., composite of loneliness, boredom, and economic concerns). Moreover, susceptibility and severity were linked to symptoms of anxiety but not depression. In contrast, within the higher risk group, both, quarantine costs and severity, predicted higher symptoms of anxiety and depression. However, perceived severity clearly showed stronger effects. This finding seems reasonable since people at higher (vs. lower) risk scored significantly higher in perceived severity.

#### Self-Isolation

In general, results show that women not only reported higher levels of distress but also less discrepancy to self-isolate. Men showed the opposite pattern, namely, lower levels of distress but greater discrepancy to self-isolate. It is important to note, however, that in this study the link between distress and intention to self-isolate is not clear enough to establish a causal relationship.

Beyond gender differences, the most important finding concerning self-isolation refers to the fact that lower and higher risk groups showed a similar pattern of results. Concretely, perceiving more quarantine benefits (i.e., sense of effectiveness and solidarity) and more self-efficacy (i.e., feeling capable of withstanding the quarantine for uncertain time) predicted less discrepancy to self-isolate.

#### Resilience and Dispositional Optimism

Finally, two important findings can be highlighted with regard to resilience and optimism. The first finding showed that, within the group at lower risk, resilience had the strongest effect against distress, symptoms of anxiety, and depression. In contrast, optimism did not show significant effects. Interestingly, this pattern of effects was reversed within the group at higher risk. In this group, dispositional optimism was the stronger predictor against distress, symptoms of anxiety, and, specially, symptoms of depression. Resilience did not show significant effects within this group. This finding suggests that people at higher risk might regulate states of distress by focusing more on positive future outcomes than on the actual moment.

The second finding reveals that resilience did not show an effect regarding the intention to self-isolate whilst dispositional optimism did. Specifically, optimism confirmed the tendency showed in the correlation analyses: Higher levels of optimism predicted higher discrepancy to self-isolate in both risk groups (note that this result is linked to the data collection period of the current study, namely, 15th April and 3rd May 2020).

## Discussion

The goal of this study was to investigate the influence of resilience and dispositional optimism on, first, emotional distress and, second, intention to self-isolate, in the particular context of the quarantine imposed in Spain during the first COVID-19 wave. Additionally, potential differences between groups at lower and higher COVID-19 risk were also explored.

The Health Belief Model (HBM; Rosenstock, 1974) was used as a suitable theoretical framework to investigate such effects (cf. Costa, 2020). Basically, the HBM assumes that the implementation of a preventive behavior (or intention to implement it; cf. Fishbein et al., 2001) requires assessing the perceived health threat and the costs/benefits associated with that preventive behavior. In this study, quarantine benefits were conceptualized as the perceived effectiveness and solidary contribution to self-isolate in line with the quarantine protocol (Webster et al., 2020). Quarantine costs were conceptualized as a composite of perceived loneliness, boredom, and economic concerns (cf. Brooks et al., 2020). Because emotional responses have only been scarcely investigated within the framework of the HBM (e.g., health information-seeking during the COVID-19 outbreak as one possible emotional response; e.g., Barattucci et al., 2020) investigating distress together with resilience and optimism was considered as an extension of the model.

In the following, the four most important findings of the study are presented and discussed.

First, people at higher risk (i.e., participants over 60 years old and/or with a chronic health condition) reported higher perceived severity. Moreover, perceived severity showed up to be the most predictive factor of emotional distress, and symptoms of anxiety and depression, within this group. In contrast, people at lower risk were more distressed by the perceived quarantine costs, which in the current study were conceptualized as a composite of perceived boredom, loneliness, and economic concerns. Therefore, it could be interpreted that two main factors predicted distress, in general, and also symptoms of anxiety and depression. One factor is linked to the health threat (i.e., perceived severity), which primarily affected people at higher risk, and the other factor is linked to the perceived “psychosocial” costs of the quarantine, which affected people at lower risk to a larger extent.

Second, contrary to what it could have been reasonable to expect, quarantine costs were not directly related to the intention to self-isolate. Instead, the results confirm prior findings, indicating that perceived benefits of preventive behaviors (quarantine benefits in this case) are one of the most important predictors of the implementation of preventive behaviors or the intention to do so (Clark et al., 2020).

Third, beyond perceived quarantine benefits, perceived self-efficacy (conceptualized as the perceived capability of withstanding the quarantine for uncertain time) showed up to be an important predictor of the intention to self-isolate in both, groups at lower and higher risk. Self-efficacy also showed to be a protective factor against distress but only within the lower risk group. In general, these findings are in line with prior studies, supporting the role of self-efficacy with regard to mental well-being (e.g., Yildirim and Güler, 2020) and the compliance with COVID-19 preventive behaviors (Chong et al., 2020).

Fourth, one of the most important findings in this study is the asymmetrical result pattern of the higher and lower risk groups with regard to the influence of resilience and optimism on emotional distress. To be more precise, the groups did not show significant differences regarding these variables. However, resilience predicted lower levels of distress within the lower risk group whereas dispositional optimism did so within the higher risk group. It is unclear why this asymmetrical pattern arose. However, a tentative explanation for this asymmetry is that people at lower risk might have focused on coping mechanisms related to overcoming the adverse quarantine effects in the present moment. In this regard, people more resilient and feeling capable of withstanding the quarantine were those showing lower levels of distress. On the other hand, people at higher risk, who in general felt more threatened by the severe consequences of getting infected, might have coped with emotional distress, in part, by feeling optimistic with regard to the positive future outcomes linked to the pandemic (e.g., “I won't contract COVID-19”).

At the same time, dispositional optimism also predicted a higher discrepancy (less intention) to self-isolate within both groups. It is important to note that we linked “discrepancy” with the need of leaving home more than strictly necessary during the quarantine. One potential explanation relates to the phenomenon of optimistic bias. The idea of optimistic bias is to underestimate the chances of experiencing negative events in comparison to peers of similar age and gender (Weinstein, 1980). In this regard, it has been shown that, in the context of the COVID-19 pandemic, dispositional optimists scored higher in optimistic bias (Monzani et al., 2021). Although we did not measure optimistic bias directly, this rationale is congruent with the fact that in the present study dispositional optimism correlated negatively with the susceptibility of getting infected by COVID-19 (see Table 4). In line with this reasoning, for some people, being too optimistic could have “relaxed” the perception of getting infected and, in turn, lead to manifest a greater intention of leaving home more than strictly necessary.

Our findings that people at higher risk show discrepancies (less intention) with preventive behaviors such as self-isolating during a pandemic quarantine seem contradictory, as also reported by Daoust (2020). A tentative explanation is that people at higher risk might have already preventive health behaviors which required special restrictions (e.g., type of diets, exercise, hospital analyses). In this case, the quarantine added further restrictions to an already restrictive lifestyle in health terms, which might be too much to cope with. The discrepancy with such preventive health behaviors within the higher risk group could also been explained by optimism. Since the adaptive sense of controllability is at the core of optimistic bias (cf. Ruthig et al., 2007), it is plausible that more optimistic people at higher risk experienced a higher sense of controllability of their own health, thus, leading to lower the perceived risks.

Another explanation refers to the state of the pandemic during the study. When the data was collected, the first COVID-19 wave in Spain was almost at its end (see Figure 1). The reported new cases and deaths were decreasing every day. It cannot be ruled out that, at least for some people at higher risk, optimism was linked to the positive expectations of being able to go outside (and thus having one restriction less if we follow the rationale that higher risk people have more restrictions), which could also explain the positive impact on mental well-being.

### Limitations

The majority of our participants were women. Therefore, there should be caution when generalizing the results to both genders. More specifically, our study showed that women (vs. men) reported greater levels of distress. This is in line with other studies investigating the psychological impact of the lockdown in Spain (e.g., Domínguez-Salas et al., 2020; Rodríguez-Rey et al., 2020) and other countries (Wang et al., 2020; Xiong et al., 2020).

Moreover, other variables such as social activity, living conditions (e.g., living alone or with the family), as well as, health-related coping styles (e.g., eating behavior; Torres and Nowson, 2007) could have been important in the perception of Covid-19 and stress management, particularly in women (e.g., Mattioli et al., 2020).

With regard to our measurement instrument, our brief *ad-hoc* questionnaire was designed to give an overview of the different HBM factors. Thus, we conceptualized quarantine costs as a composite of boredom, loneliness, and economic concerns. It can, however, be interesting to investigate the impact of each of the elements of quarantine costs on mental health and preventive behaviors during the Covid-19 pandemic. For example, loneliness alone has been an important predictor of symptoms of anxiety and depression during the COVID-19 pandemic (e.g., Palgi et al., 2020). Similarly, the two items assessing social pressure and self-efficacy within the HBM were designed to be context-specific and it might be fruitful to inspect these variables in more detail (e.g., by adding items).

Another limitation might be that it cannot be ruled out that responses to the intention to self-isolate were influenced by social desirability.

Additionally, differences between countries regarding the management of the pandemic, particularly at the initial stage can suppose also a limitation with regard to the generalization of the findings of this study. Nonetheless, it should be noted that some of the findings in this study are in line with prior research from other countries (e.g., effects of perceived benefits on preventive behavior; e.g., Clark et al., 2020).

### Conclusions

In the short term, the findings of this study might contribute to better understanding the people's experiences during the COVID-19 pandemic and the related mitigation measures, which can lead to improving governmental initiatives, e.g., communication campaigns should prioritize information about the effectiveness of the implemented preventive behaviors rather than the costs of not implementing them, and should also be cautious in encouraging excessive optimism. In the long term, the findings contribute to understand, in more detail, the effects of resilience and dispositional optimism on cognition and emotion within the specific context of negative events, such as a pandemic outbreak.

## Data Availability Statement

The raw data supporting the conclusions of this article will be made available upon request to the authors.

## Ethics Statement

Ethical review and approval was not required for the study on human participants in accordance with the local legislation and institutional requirements. The patients/participants provided their written informed consent to participate in this study.

## Author Contributions

SC-T and SR-F conceived the original idea. SC-T, SR-F, and HG were involved in the data analyses and interpretation. SC-T and LM drafted the manuscript. DM-R, SP-C, and RB critically reviewed the work. All authors approved the final version of the manuscript and agreed to be accountable for the accuracy and integrity of the project. All authors were involved in the design of the work and data acquisition.

## Conflict of Interest

The authors declare that the research was conducted in the absence of any commercial or financial relationships that could be construed as a potential conflict of interest.
