# Supplementary material for: Influence of Resilience and Optimism on Distress and Intention to Self-Isolate: Contrasting Lower and Higher COVID-19 Illness Risk Samples From an Extended Health Belief Model
Source: Front Psychol. 2021 May 24;12:662395. doi: 10.3389/fpsyg.2021.662395 (PMC8180876; doi:10.3389/fpsyg.2021.662395)
Supplement: Supplementary file 1 [file Table_1.pdf]

Confirmatory Factor Analysis (CFA)

In CFA, several indicators estimate de fitness of the model. The most common are the Comparative Fit Index (CFI), the Tucker-Lewis index (TLI), the Root Mean Square Error of Approximation (RMSEA), and the Standardize Root Mean Square Residual (SRMR). CFI and TLI values ≥ .90 (ideally ≥ .95), RMSEA ≤ .06, and SRMR ≤ .08, indicate good model fitness. However, severe departures from normal distribution of the data can also bias the estimation of these parameters (e.g., Brown, 2015).

In this study, the items representing *quarantine benefits* showed that type of departure (see Table 1). To deal with this issue, the unweighted least squares (ULS) CFA estimator, which fits better to ordinal data and has fewer assumptions on the data structure (e.g., Koğar & Koğar, 2015), was imposed on the standardized raw responses. Data were analysed through the JASP software v0.14.1.

Results indicated a CFI = .98, TLI = .97, RMSEA = .026, and SMRM = .045. Standardized factor loadings (long arrows in supplementary Figure 1) were all statistically significant ($p \leq .002$).

Supplementary Table 1. Descriptive statistics of the ad-hoc HBM questionnaire

Items	*M* (SD)	Skewness	Kurtosis	LR *M*(SD)	HR *M*(SD)	*t*	*p*
**Susceptibility**							
Item 1	4.09 (1.76)	-.042	-.77	4.15 (1.80)	3.95 (1.65)	.99	.32
Item 2*	3.02 (1.95)	.57	-.87	2.94 (1.94)	3.22 (1.98)	-1.19	.23
**Severity**							
Item 3	4.06 (1.76)	-.074	-.78	3.97 (1.75)	4.28 (1.77)	-1.41	.16
Item 4	4.29 (1.89)	-.079	-1.06	4.06 (1.88)	4.86 (1.79)	-3.55	.001
Item 5	5.34 (1.97)	-.97	-.35	5.18 (2.04)	5.74 (1.75)	-2.52	.012
**Q. Benefits**							
Item 6	6.58 (1.01)	-3.31	12.53	6.56 (1.06)	6.62 (.88)	-.47	.64
Item 7	6.62 (.87)	-3.05	11.18	6.61 (.91)	6.64 (.79)	-.26	.79
**Q. Costs**							
Item 8	3.20 (1.98)	1.99	-1.13	3.09 (1.97)	3.47 (2.02)	-1.55	.12
Item 9	3.86 (1.44)	1.44	-.172	3.85 (1.43)	3.88 (1.46)	-1.71	.86
Item 10	4.41 (2.09)	-.39	-1.15	4.59 (2.09)	3.97 (2.03)	2.46	.014

(*) Inverted Item. Skewness |S| ≥ 3 and Kurtosis ≥ 7 |K| are considered as departures of normal distribution (Kim, 2013). LR (Lower illness risk) HR (Hiher illness risk)

KOĞAR, H., & KOĞAR, E. Y. (2015). Comparison of different estimation methods for categorical and ordinal data in confirmatory factor analysis. *Eğitimde ve Psikolojide Ölçme ve Değerlendirme Dergisi*, *6*(2), 351-364
