# Supplementary material for: Influence of Resilience and Optimism on Distress and Intention to Self-Isolate: Contrasting Lower and Higher COVID-19 Illness Risk Samples From an Extended Health Belief Model
Source: Front Psychol. 2021 May 24;12:662395. doi: 10.3389/fpsyg.2021.662395 (PMC8180876; doi:10.3389/fpsyg.2021.662395)
Supplement: Supplementary file 2 [file Table_2.pdf]

Supplementary Table 2. Generalized regression analyes of Symptoms of Depression and Symptoms of Anxiety

**S. Depression**	Total sample *Pseudo* R^2 = .291			Lower Illness Risk *Pseudo* R^2 = .316			Higher Illness Risk *Pseudo* R^2 = .453		
	b	95% CI	$b_{\%}$ (ΔR^2)	*b*	95% CI	$b_{\%}$ (ΔR^2)	*b*	95% CI	$b_{\%}$ (ΔR^2)
Gender	-.204	(.049, .358)**	22.6% (.030)**	-.106	(-.051, .263)	-	-.306	(.030, .582)*	35.8% (.061)*
Age	-.102	(-.179, -.025)**	10.7% (.047)**	-.079	(-.158, -.001)*	8.2% (.062)**	-.259	(-.415, -.101)**	29.5% (.099)**
Susceptibility	.049	(-.036, .133)	-	.019	(-.064, .101)	-	.159	(-.062, .379)	-
Severity	.044	(-.041, .130)	-	-.016	(-.097, .065)	-	.167	(.047, .287)**	18.1% (.115)*
Q. Costs	.176	(.101, .251)**	19.3% (.144)**	.203	(.131, .275)**	22.5% (.162)**	.238	(.088, .388)**	26.9% (.075)*
Q. Benefits	-.007	(-.064, .050)	-	.006	(-.037, .049)	-	-.050	(-.139, .040)	-
Social Pressure	.025	(-.055, .105)	-	.054	(-.030, .137)	-	-.129	(-.349, .091)	-
Self-Efficacy	-.079	(-.155, -.004)*	8.2% (.028)†	-.123	(-.199, -.046)**	12.9% (.054)†	-.013	(-.168, .141)	-
Resilience	-.160	(-.218, -.102)**	17.4% (.075)**	-.151	(-.211, -.091)**	16.3% (.079)**	-.125	(-.253, .002)†	-
D. Optimism	-.034	(-.118, .050)	-	.017	(-.057, .091)	-	-.267	(-.450, -.085)**	30.7% (.209)*

**S. Anxiety**	*Pseudo* R^2 = .316			*Pseudo* R^2 = .332			*Pseudo* R^2 = .496		
	b	95% CI	$b_{\%}$ (ΔR^2)	*b*	95% CI	$b_{\%}$ (ΔR^2)	*b*	95% CI	$b_{\%}$ (ΔR^2)
Gender	-.253	(.086, .419)**	28.7% (.042)**	-.302	(.101, .502)**	35.2% (.052)**	.010	(-.324, .304)	-
Age	-.042	(-.113, .029)	-	.013	(-.066, .093)	-	-.256	(-.383, -.128)**	29.0% (.190)**
Susceptibility	.091	(.022, .161)**	9.6% (.045)**	.115	(.040, .191)**	12.2% (.057)**	.077	(-.043, .197)	-
Severity	.189	(.106, .272)**	20.8% (.122)**	.104	(.018, .189)*	10.9% (.065)**	.266	(.160, .372)**	30.4% (.235)**
Q. Costs	.134	(.062, .205)**	14.3% (.074)**	.174	(.093, .256)**	19.0% (.121)**	.102	(.012, .192)*	10.8% (.025)
Q. Benefits	-.009	(-.066, -.049)	-	-.020	(-.078, .037)	-	.122	(-.044, .289)	-
Social Pressure	.023	(-.055, .100)	-	.030	(-.070, .129)	-	-.072	(-.214, .070)	-
Self-Efficacy	-.048	(-.117, .022)	-	-.084	(-.167, -.002)*	8.8% (.029)	-.001	(-.110, .107)**	-
Resilience	-.140	(-.195, -.086)**	15.1% (.079)**	-.132	(-.197, -.067)*	14.1% (.059)**	-.120	(-.240, .001)†	-
D. Optimism	-.019	(-.086, .049)	-	.093	(-.043, .094)	-	-.128	(-.246, -.011)*	13.6% (.159)
