# Supplementary figures and images for: Influence of Resilience and Optimism on Distress and Intention to Self-Isolate: Contrasting Lower and Higher COVID-19 Illness Risk Samples From an Extended Health Belief Model
Source: Front Psychol. 2021 May 24;12:662395. doi: 10.3389/fpsyg.2021.662395 (PMC8180876; doi:10.3389/fpsyg.2021.662395)

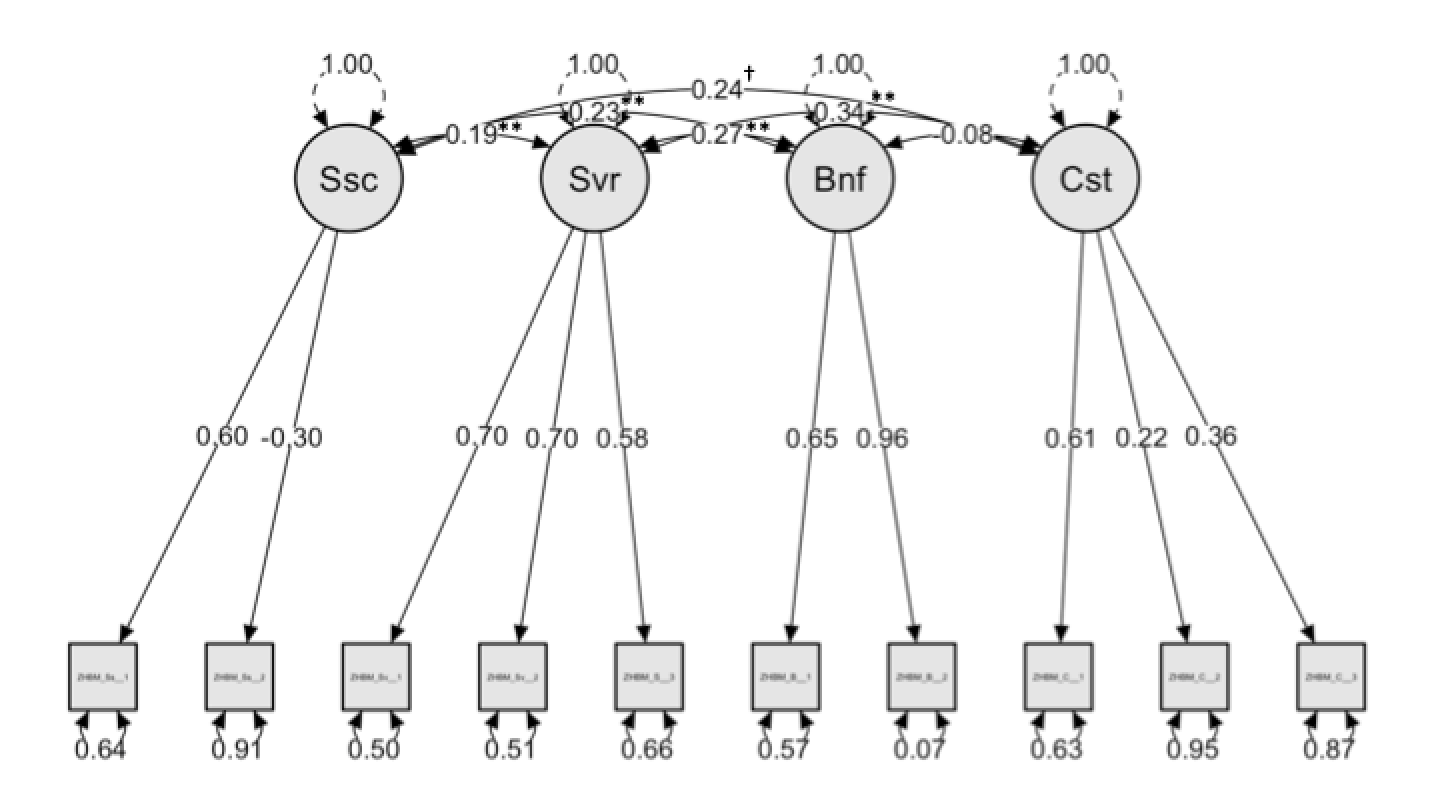

Supplement: Supplementary file 3 [file Image_1.tif]
